# Impact of patient-specific three-dimensional reconstruction on surgical planning and perioperative outcomes in early-stage lung cancer: a randomised clinical trial

**DOI:** 10.3389/fonc.2026.1887152

**Published:** 2026-08-21

**Authors:** Li Hang Liao, Zhang Yong Ren, Xin Lian Yan, Ming Hai Chen, Xu Zhu, Xiao Jun Du

**Affiliations:** Department of Thoracic Surgery, The Affiliated Hospital of Guizhou Medical University, Guiyang, China

**Keywords:** NSCLC, preoperative planning, randomised clinical trial, segmentectomy, surgical quality, three-dimensional reconstruction, uniportal VATS

## Abstract

**Background:**

Anatomical segmentectomy requires precise identification of the segmental bronchi and vessels; however, conventional two-dimensional (2D) CT provides limited visualisation of their three-dimensional (3D) anatomical relationships and variations.

**Methods:**

In this prospective randomised controlled trial, patients with early-stage non-small cell lung cancer (NSCLC) who were scheduled to undergo uniportal thoracoscopic anatomical segmentectomy were assigned to either the 2D CT group or the 3D reconstruction group. The trial was registered with the Chinese Clinical Trial Registry before patient enrolment (ChiCTR2500104962; registered 26 June 2025). The primary outcome was key-phase operative time, defined as the interval from the start of segmental hilar dissection to completion of segmentectomy. Total operative time, defined as the interval from skin incision to closure, was assessed as an additional time-based outcome. Secondary outcomes included intraoperative unexpected events, attainment of adequate pathological margins, and surgeon-reported experience. Prespecified subgroup analyses were conducted according to surgeon seniority and segmentectomy complexity. Surgeon-reported experience was assessed using structured questionnaires.

**Results:**

A total of 144 patients were included. Compared with the 2D CT group, the 3D group demonstrated markedly reduced key-phase operative time (75.78 ± 24.44 min vs 95.74 ± 31.43, P < 0.001), fewer intraoperative unexpected events (6.9% vs 30.6%, P = 0.001), and a higher rate of achieving safe surgical margins (84.7% vs 66.7%, P = 0.016). No statistically significant differences were observed in total operative time, postoperative complications, or other perioperative outcomes between the two groups (P > 0.05). In contrast, surgeons in the 3D group reported superior preoperative anatomical visualization, enhanced intraoperative confidence, and reduced perceived stress levels (all P < 0.001). Notably, the reduction in key-phase operative time was more prominent among surgeons with less clinical experience (P = 0.018).

**Conclusion:**

Preoperative 3D reconstruction shortened key-phase operative time, reduced intraoperative unexpected events, improved pathological margin attainment, and was associated with more favourable surgeon-reported experience.

## 1 Introduction

For patients with peripheral early-stage NSCLC measuring 2 cm or less, anatomical segmentectomy has been shown to be oncologically non-inferior to lobectomy (1, 2). Compared with lobectomy, segmentectomy preserves more functional lung parenchyma and may therefore result in better postoperative pulmonary function and quality of life. Nevertheless, segmentectomy remains technically demanding. Successful segmentectomy requires accurate localisation of the tumour, precise delineation of segmental anatomy, and an understanding of the complex three-dimensional (3D) relationships among the bronchi, pulmonary arteries, and pulmonary veins (3, 4). Unlike lobectomy, which is generally performed in a relatively spacious operative field, segmentectomy is often undertaken in a more confined space with more intricate anatomical planes. Common but easily overlooked anatomical variations further increase this complexity (5). These contemporary findings should be considered alongside the historical Lung Cancer Study Group trial and subsequent Japanese studies supporting selected sublobar resection for ground-glass-dominant tumours (6–8).

Preoperative three-dimensional (3D) reconstruction based on thin-slice CT has increasingly been adopted to facilitate thoracoscopic segmentectomy by providing intuitive, patient-specific visualisation of bronchovascular anatomy and tumour location (9–12). This technique may reveal anatomical variations that are difficult to appreciate on conventional two-dimensional (2D) imaging, thereby reducing unexpected vascular injury and other anatomy-related intraoperative events (13). Retrospective studies have suggested that 3D-assisted segmentectomy may shorten operative time, reduce intraoperative resource use, and potentially improve perioperative safety (14–16). However, the available evidence remains heterogeneous, and at least one randomised trial reported no significant difference in total operative time (17). Earlier clinical series have shown that patient-specific 3D CT reconstruction can assist with tumour localisation, bronchovascular mapping, and preoperative margin assessment before anatomical segmentectomy important gaps remain in the current literature.

First, most published studies are retrospective and are therefore susceptible to selection bias. Second, previous studies have focused primarily on patient-centred perioperative outcomes, whereas surgeons’ intraoperative experience has rarely been evaluated in a structured manner.

Consequently, it remains unclear whether the ‘visual navigation’ provided by 3D reconstruction translates into greater operative confidence, clearer intraoperative decision-making, and lower perceived stress during critical surgical steps. We therefore conducted a prospective randomised controlled trial to evaluate the clinical utility of preoperative 3D CT reconstruction in uniportal thoracoscopic anatomical segmentectomy for early-stage NSCLC.

## 2 Methods

### 2.1 Study design and oversight

This prospective, single-center, parallel-group randomised clinical trial was conducted at the Department of Thoracic Surgery, The Affiliated Hospital of Guizhou Medical University, from July 2025 to January 2026, with short-term follow-up for 2 months after surgery. The trial protocol was approved by the Ethics Committee of The Affiliated Hospital of Guizhou Medical University (approval No. 2024-473), and all participants provided written informed consent before enrollment. The trial was registered with the Chinese Clinical Trial Registry before patient enrollment (ChiCTR2500104962; registered June 26, 2025). The study is reported according to the CONSORT 2025 reporting guideline (20).

### 2.2 Participants

Eligible patients were required to have a solitary pulmonary nodule measuring 2 cm or less on chest CT, radiological findings consistent with clinical stage IA NSCLC, a lesion confined to a single pulmonary segment, and eligibility for anatomical segmentectomy on the basis of pulmonary function testing and imaging. Prespecified radiological indications included a part-solid or pure ground-glass nodule measuring 2 cm or less, a consolidation-to-tumour ratio of less than 50%, and a location within the peripheral two-thirds of the lung parenchyma (21, 22).

Lesions adjacent to the pleura or an intersegmental fissure were excluded. Additional exclusion criteria included previous ipsilateral thoracic surgery or severe ipsilateral thoracic trauma, postoperative pathological findings of small-cell lung cancer, carcinoid tumour, or metastatic disease, and imaging evidence of another primary lesion requiring wedge resection, multilobar resection, or subsegmentectomy.

### 2.3 Randomisation and masking

Eligible patients were randomly assigned in a 1:1 ratio to the 3D reconstruction group or the 2D CT control group using a computer-generated allocation sequence. Treatment assignments were concealed in sequentially numbered, sealed, opaque envelopes and opened after enrollment. Surgeons and patients could not be blinded because the intervention required preoperative imaging review. Perioperative outcomes and complications were adjudicated by independent investigators who were not involved in the operations whenever possible, and who applied prespecified operational definitions. Randomisation was performed at the patient level and was not stratified by individual operating surgeon.

### 2.4 3D reconstruction workflow

All patients underwent thin-slice high-resolution CT with a slice thickness of 1.0–2.0 mm. For patients assigned to the 3D reconstruction group, CT data in Digital Imaging and Communications in Medicine (DICOM) format were imported into dedicated reconstruction software (InferVision Research Edition H4.7.0; National Medical Products Administration registration No. 20233212016) to generate patient-specific models of the bronchial tree, pulmonary arteries, pulmonary veins, and target lesion. Before surgery, the operating team reviewed the models to identify the target segmental bronchovascular structures, anatomical variations, the anticipated intersegmental plane, and the spatial relationship between the lesion and the planned parenchymal transection plane, and to assess the virtual surgical margin. The models were generally completed within 24 hours before surgery, and each reconstruction required approximately 30 minutes. During surgery, the models could be viewed interactively on a computer and used as an anatomical reference. In the 2D CT group, preoperative planning was based solely on conventional multiplanar CT images. All other perioperative management procedures were standardised between the two groups.

### 2.5 Surgical procedure

All procedures were performed via uniportal VATS and followed standardized principles for anatomical segmentectomy. The segmental pulmonary artery, vein, and bronchus of the target segment were sequentially dissected and divided. After bronchial division, the inflation-deflation method was used to identify the intersegmental plane, and the segment was resected with a linear stapler. The specimen underwent intraoperative frozen-section assessment of histology and margin status. Completion lobectomy was performed for a positive margin. If the margin was below the prespecified safety threshold (<2 cm or less than the maximum tumor diameter), additional wedge resection or lobectomy was performed to obtain an adequate oncologic margin.

### 2.6 Outcomes

Baseline characteristics and perioperative outcomes were collected for both groups. The operating surgeon completed the questionnaire within 2 hours after surgery. Intraoperative variables, including pleural adhesions, fissure development, and surgeon seniority, were recorded; operational definitions are provided in **Supplementary Material 1.**

The primary endpoint was key-phase operative time, defined as the interval from the commencement of anatomical segmental dissection—marked by the start of identification and management of the target segmental vessels—to completion of segmentectomy. Time spent on intraoperative frozen-section assessment, lymph node dissection, and chest closure was excluded. The duration was recorded intraoperatively by a study investigator using a standardised time-recording form and was subsequently verified by review of the operative video. The same predefined anatomical landmarks and timing procedures were applied consistently in both groups to improve measurement consistency and reproducibility. Total operative time, defined as the interval from skin incision to skin closure, was analysed as an additional time-based endpoint. Secondary outcomes included intraoperative blood loss, chest tube duration, postoperative length of stay, intraoperative unexpected events, 30-day postoperative complications, and adequate pathological margin attainment. Intraoperative unexpected events were prespecified as vascular injury, erroneous division of a vessel or bronchus, unplanned additional resection because of failed lesion localisation or an anticipated inadequate margin, and conversion to lobectomy. Postoperative complications included prolonged air leak lasting more than 5 days, pulmonary infection, chylothorax, and reoperation. Adequate pathological margin attainment was defined as a closest pathological margin of at least 2 cm (23). For consistency, the regression models were parameterised to assess the risk of margin inadequacy.

The operating surgeon completed a trial-specific, self-administered questionnaire within 2 hours after surgery. The questionnaire assessed three domains: preoperative anatomical understanding and planning, confidence during key operative steps, and perceived intraoperative stress and workload. Although the questionnaire underwent internal psychometric assessment, it has not been externally validated in an independent population. Because surgeons could not be blinded to group allocation, the questionnaire outcomes were considered subjective and exploratory. Questionnaire data were unavailable for some randomised procedures; therefore, these analyses were conducted using an available-case approach (**Supplementary Material 1**).

### 2.7 Sample size estimation

Before trial initiation, pilot data from 42 eligible patients treated at our centre were reviewed. Because key-phase operative time had not been fully standardised during the pilot phase, the pooled standard deviation of total operative time was used as a conservative estimate for the sample size calculation. The standard deviations in the two pilot cohorts were 48.4 and 46.3 minutes, yielding a pooled standard deviation of approximately 47.3 minutes. The minimal clinically important difference was prespecified as 24 minutes. Assuming a two-sided alpha level of 0.05 and 80% power, 60 patients were required in each group. The target sample size was increased to 144 patients (72 per group) to allow for potential attrition and to improve precision.

## 3 Statistical analysis

Perioperative outcomes were analysed according to the intention-to-treat principle, whereas questionnaire data were analysed using an available-case approach. Continuous variables are presented as mean ± standard deviation or median (interquartile range), as appropriate, and were compared using the independent-samples t-test or a non-parametric test. Categorical variables are presented as counts and percentages and were compared using the chi-squared test or Fisher’s exact test, as appropriate. For binary outcomes, robust Poisson regression was used to estimate risk ratios with 95% confidence intervals. Prespecified subgroup analyses were performed according to surgeon seniority and segmentectomy complexity. Key-phase operative time was analysed using linear regression with interaction testing. Because risk-ratio models did not converge in some subgroup analyses of binary outcomes, logistic regression was used for these exploratory analyses, and odds ratios with 95% confidence intervals were reported. All tests were two-sided, and P < 0.05 was considered statistically significant. Statistical analyses were performed using SPSS version 31.0 and R version 4.2.3.

## 4 Results

### 4.1 Participant enrollment and baseline characteristics

**Figure 1:**
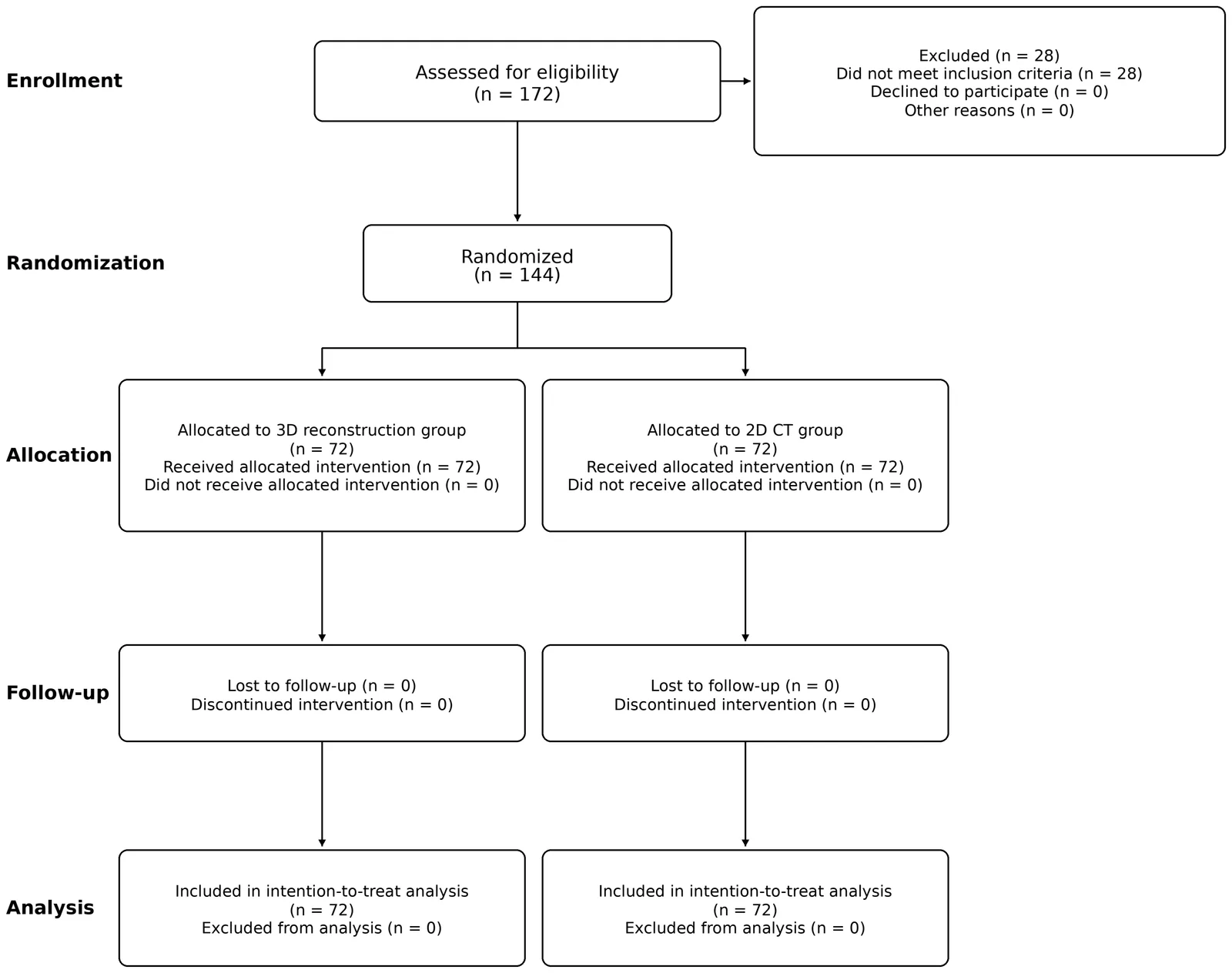
Participant flow through the randomised clinical trial.

Between July 2025 and January 2026, 172 patients were screened and 144 eligible patients were randomised, with 72 assigned to the 3D reconstruction group and 72 assigned to the 2D CT group (**Figure 1**). All randomised patients were included in the intention-to-treat analysis. Baseline demographic, clinical, and surgical characteristics are presented in **Table 1**. No perioperative deaths occurred.

**Table 1:** Baseline characteristics.

Baseline characteristics	2D CT group (n = 72)	3D reconstruction group (n = 72)
Age, mean (SD), years	58.9 (11.2)	58.7 (12.3)
Maximum tumor size, mean (SD), cm	1.30 (0.41)	1.27 (0.57)
Sex, n (%)		
Male	35 (48.6)	38 (52.8)
Female	37 (51.4)	34 (47.2)
Smoking status, n (%)		
Never	43 (59.7)	52 (72.2)
Current	29 (40.3)	20 (27.8)
Segmentectomy complexity, n (%)		
Simple	26 (36.1)	30 (41.7)
Complex	46 (63.9)	42 (58.3)
Comorbidity, n (%)		
No	47 (65.3)	48 (66.7)
Yes	25 (34.7)	24 (33.3)
Surgeon seniority, n (%)		
Junior	18 (25.0)	28 (38.9)
Senior	54 (75.0)	44 (61.1)
Pleural adhesions, n (%)		
Present	55 (76.4)	56 (77.8)
Absent	17 (23.6)	16 (22.2)
Fissure development, n (%)		
Poorly developed	25 (34.7)	15 (20.8)
Well developed	47 (65.3)	57 (79.2)

### 4.2 Perioperative outcomes

**Figure 2:**
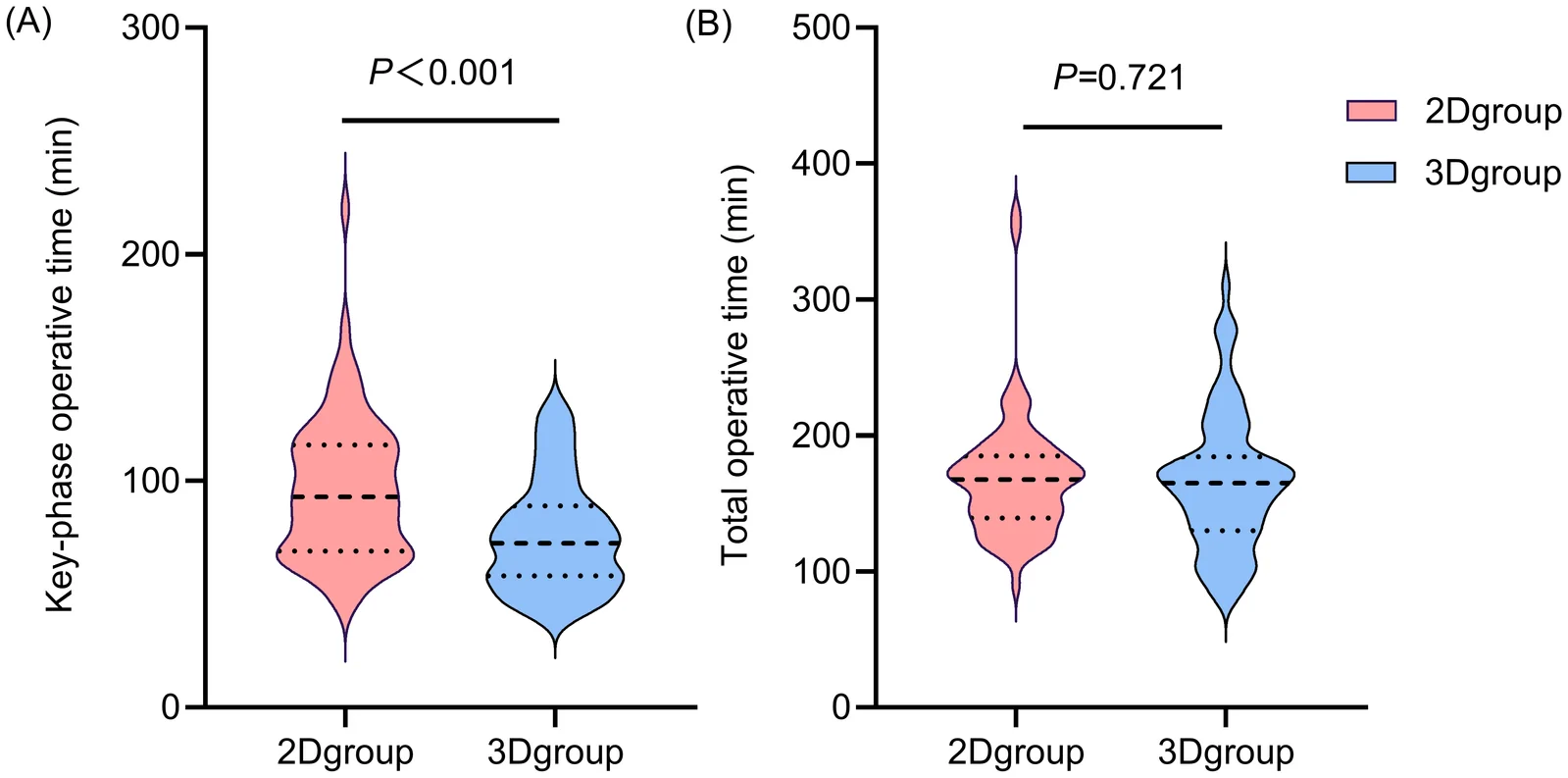
Comparison of operative times between the 3D reconstruction and 2D CT groups. **(A)** Key-phase operative time. **(B)** Total operative time.

All procedures were completed successfully, and no perioperative deaths occurred. Key-phase operative time was significantly shorter in the 3D reconstruction group than in the 2D CT group (75.78 ± 24.44 vs 95.74 ± 31.43 min; P < 0.001), whereas total operative time was similar between the groups (165.76 ± 49.74 vs 168.48 ± 44.75 min; P = 0.721) (**Figure 2**).

**Figure 3:**
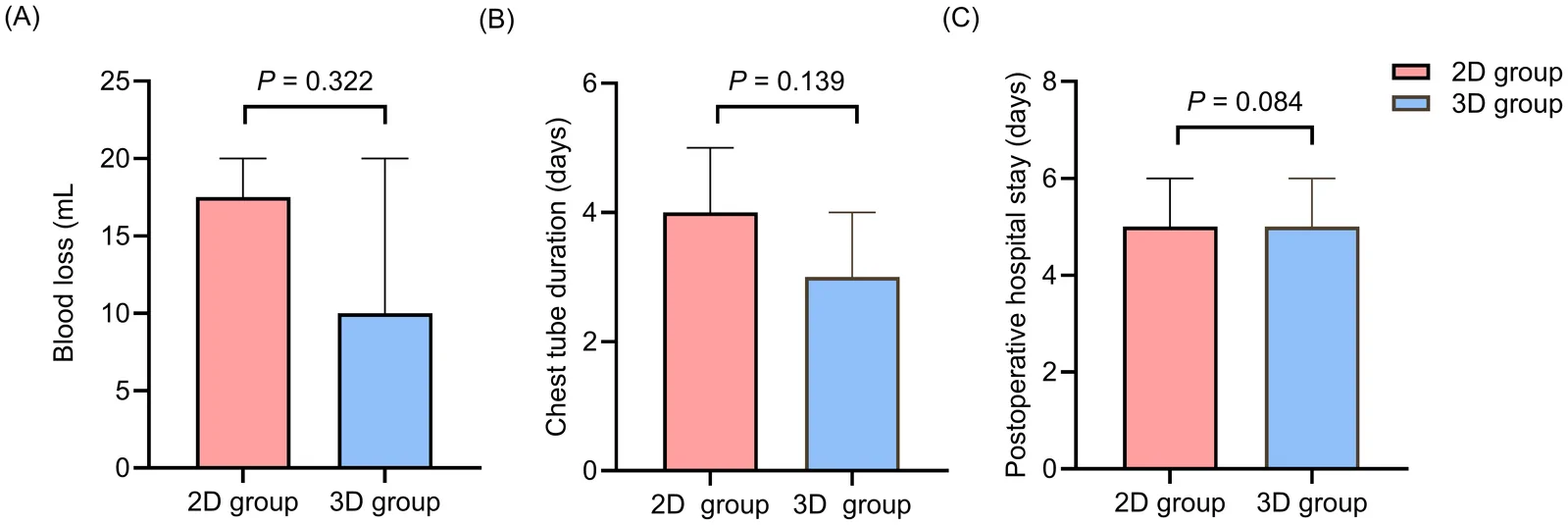
Comparison of perioperative outcomes between the 3D reconstruction and 2D CT groups. **(A)** Intraoperative blood loss. **(B)** Chest tube duration. **(C)** Postoperative length of stay.

Median intraoperative blood loss was 10.0 mL in the 3D reconstruction group and 17.5 mL in the 2D CT group (interquartile range [IQR], 10–20 mL in both groups; P = 0.322). Median chest tube duration was 3 days in the 3D reconstruction group and 4 days in the 2D CT group (IQR, 2.25–4.0 vs 3–5 days; P = 0.139). Median postoperative length of stay was 5 days in both groups (IQR, 4–6 vs 5–6 days; P = 0.084) (**Figure 3**).

**Figure 4:**
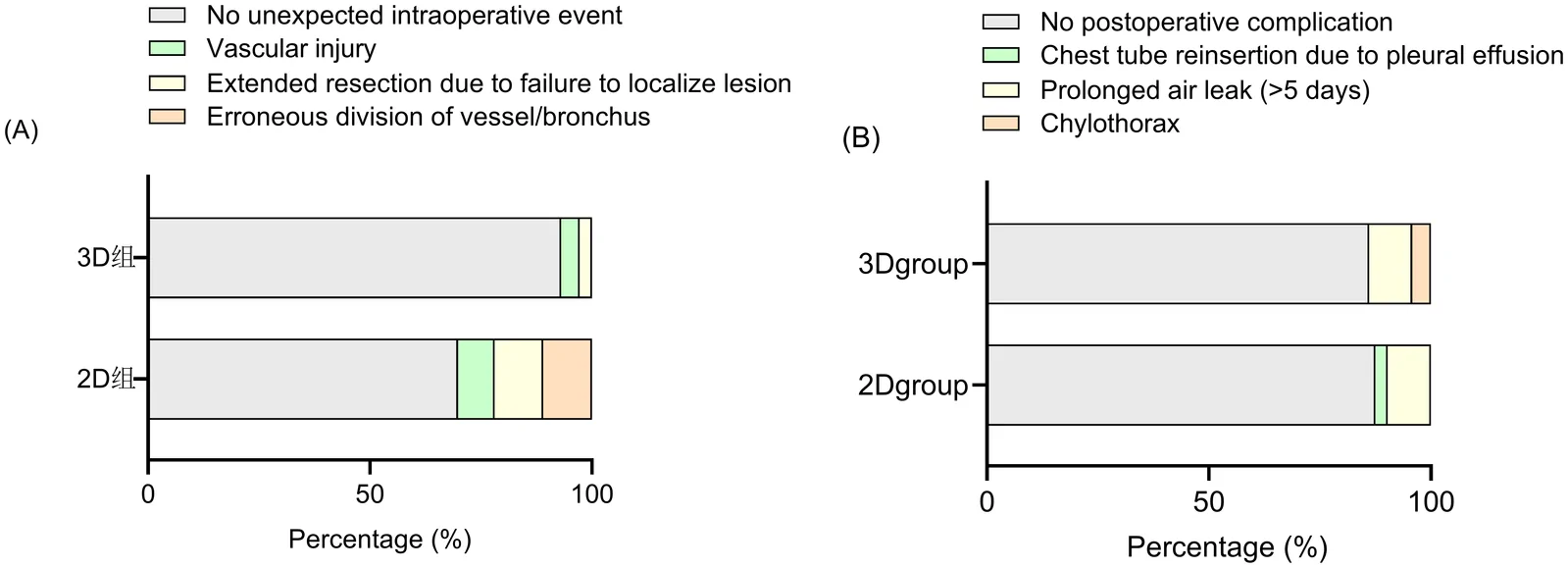
Intraoperative unexpected events and postoperative complications in the 3D reconstruction and 2D CT groups. **(A)** Intraoperative unexpected events. **(B)** Postoperative complications.

**Figure 5:**
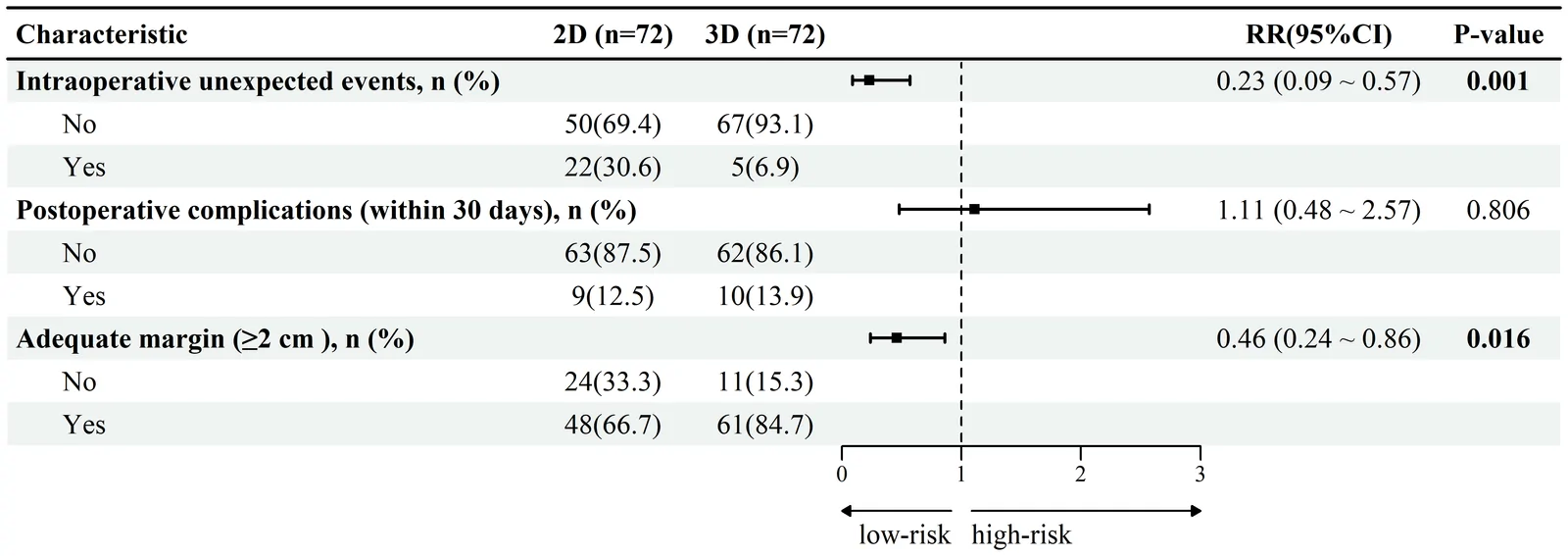
Forest plot comparing intraoperative unexpected events, 30-day postoperative complications, and adequate pathological margin attainment between the 3D reconstruction and 2D CT groups. For margin status, the regression model was parameterised with margin inadequacy as the outcome; therefore, an RR < 1 favours the 3D reconstruction group.

The 3D reconstruction group had fewer intraoperative unexpected events than the 2D CT group (6.9% vs 30.6%; RR, 0.23; 95% CI, 0.09–0.57; P = 0.001). In the 2D CT group, vascular injury occurred in 6 patients (8.3%), erroneous division of a vessel or bronchus in 8 patients (11.1%), and unplanned additional resection because of unsuccessful lesion localisation or concern about an anticipated inadequate margin in 8 patients (11.1%). In the 3D reconstruction group, vascular injury occurred in 3 patients (4.2%) and unplanned additional resection in 2 patients (2.8%); no erroneous division of a vessel or bronchus was observed (**Figures 4**, **5**). Given the small number of individual events, component-level findings were considered descriptive.

A higher proportion of patients in the 3D reconstruction group achieved adequate pathological margins than in the 2D CT group (84.7% vs 66.7%; P = 0.016), corresponding to a lower risk of margin inadequacy (RR, 0.46; 95% CI, 0.24–0.86) (**Figure 5**).

Thirty-day postoperative complication rates were similar between the groups (12.5% in the 2D CT group vs 13.9% in the 3D reconstruction group; P = 0.806). In the 2D CT group, complications included pleural effusion requiring repeat drainage (n = 2) and prolonged air leak lasting more than 5 days (n = 7). In the 3D reconstruction group, complications included chylothorax (n = 3, including one case requiring reoperation) and prolonged air leak lasting more than 5 days (n = 7). No deaths occurred during the 2-month follow-up period (**Figures 4**, **5**).

### 4.3 Surgeon questionnaire analysis

**Figure 6:**
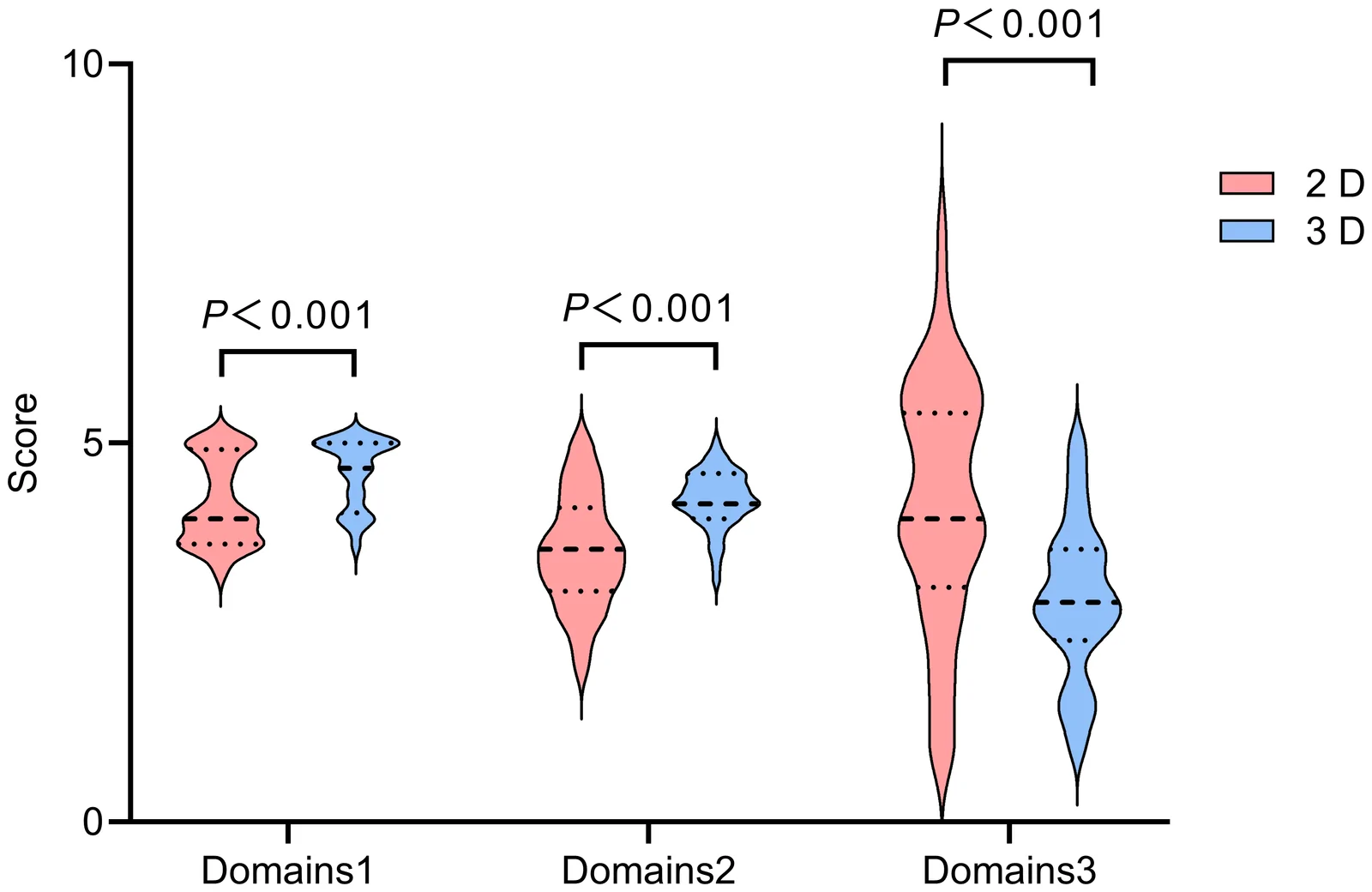
Violin plots comparing surgeon-reported experience scores between the 3D reconstruction and 2D CT groups across three questionnaire domains.Domain 1 represents preoperative anatomical understanding and planning; Domain 2 represents confidence during key operative steps; and Domain 3 represents perceived intraoperative stress and workload.

A total of 102 questionnaires were collected: 47 from the 3D reconstruction group and 55 from the 2D CT group. Compared with the 2D CT group, surgeons in the 3D reconstruction group reported greater preoperative anatomical clarity (4.65 ± 0.42 vs 4.20 ± 0.56; P < 0.001), higher intraoperative confidence (4.24 ± 0.37 vs 3.56 ± 0.74; P < 0.001), and lower perceived stress and workload (2.54 ± 0.72 vs 3.48 ± 1.31; P < 0.001). Because questionnaire data were not available for the entire randomised cohort, these findings were considered exploratory rather than confirmatory (**Figure 6**).

### 4.4 Subgroup analyses

**Figure 7:**
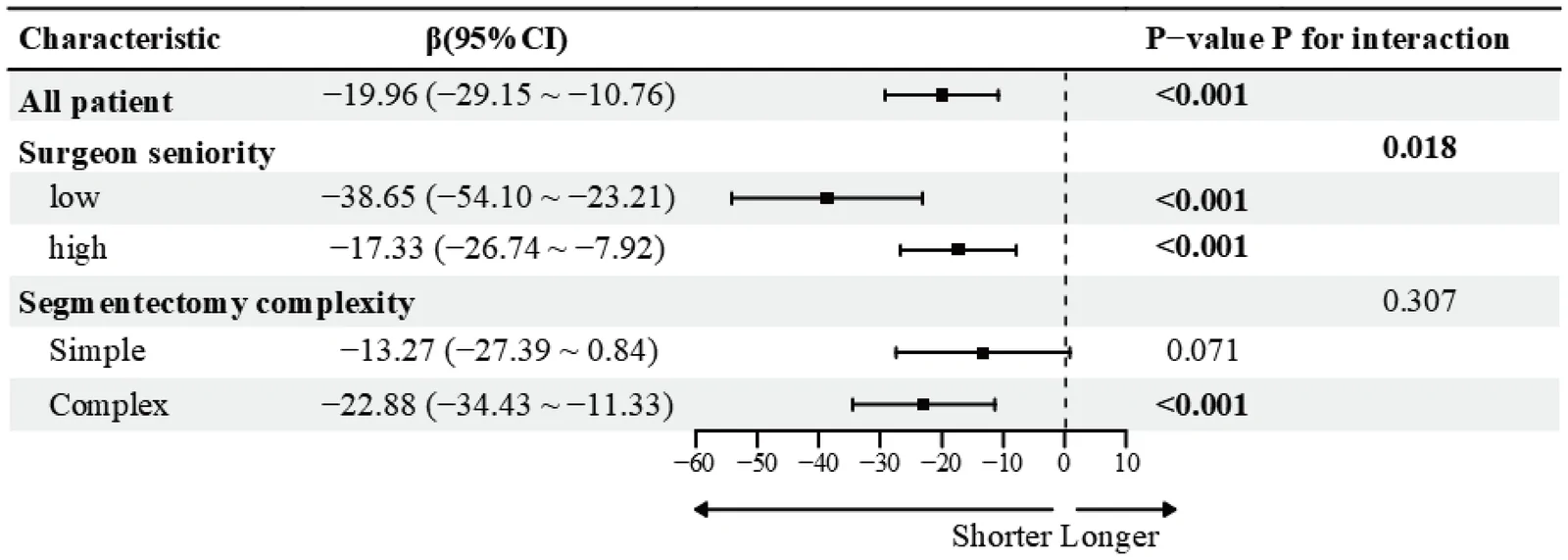
Forest plot of subgroup analyses for key-phase operative time. Effects are expressed as β coefficients (minutes) with 95% confidence intervals (CIs) estimated using linear regression, with the group contrast defined as 3D reconstruction minus 2D CT. A β < 0 indicates a shorter key-phase operative time in the 3D reconstruction group. Squares represent point estimates, and horizontal lines represent 95% CIs; the vertical dashed line at 0 indicates no between-group difference. P values indicate within-subgroup effects, whereas P values for interaction assess effect modification by surgeon seniority and segmentectomy complexity.

The direction of the effect of 3D reconstruction on key-phase operative time was consistent across the prespecified subgroups. The reduction was greater among junior surgeons than among senior surgeons (38.65 vs 17.33 min; P for interaction = 0.018). In complex segmentectomies, 3D reconstruction reduced key-phase operative time by approximately 22.88 minutes (P < 0.001), whereas the reduction in simple segmentectomies was smaller and did not reach statistical significance (approximately 13.3 minutes; P = 0.071). However, the interaction according to segmentectomy complexity was not statistically significant (P for interaction = 0.307) (**Figure 7**).

**Figure 8:**
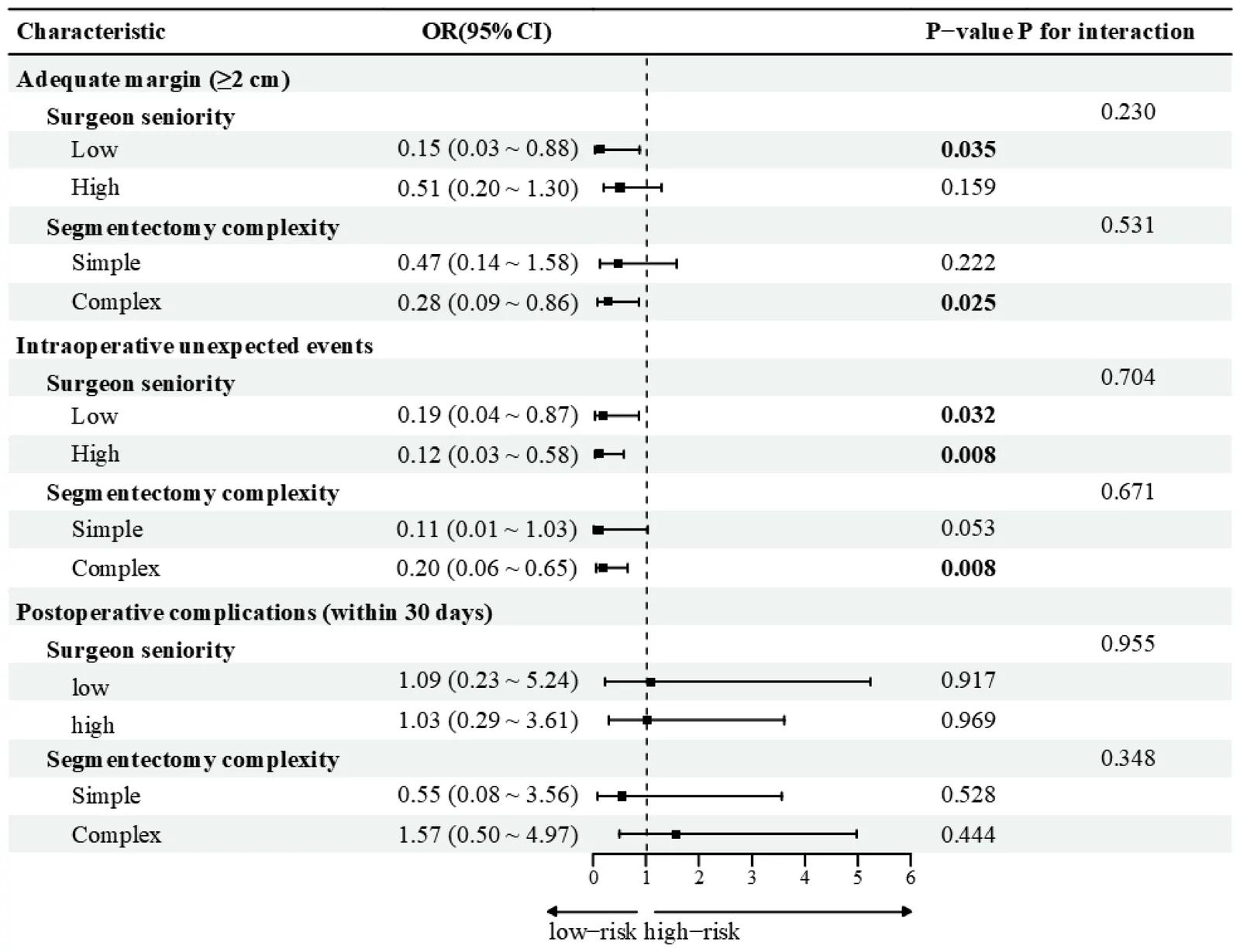
Forest plot of subgroup analyses for binary perioperative outcomes comparing the 3D reconstruction group with the conventional 2D CT group. Odds ratios (ORs) with 95% confidence intervals (CIs) were estimated using logistic regression. Subgroups were defined according to surgeon seniority (junior vs senior) and segmentectomy complexity (simple vs complex). Squares represent point estimates, and horizontal lines represent 95% CIs; the vertical dashed line at OR = 1 indicates no between-group difference. P values indicate within-subgroup effects, whereas P values for interaction test whether the group effect differs according to surgeon seniority or segmentectomy complexity. Outcomes include adequate pathological margin attainment (≥2 cm), intraoperative unexpected events, and postoperative complications within 30 days.

Across the prespecified subgroups, the direction of effect for intraoperative unexpected events and adequate pathological margin attainment was consistent with the main analysis, and most interaction tests were not statistically significant. Because postoperative complications were infrequent and the corresponding confidence intervals were wide, no significant between-group differences were observed in subgroup analyses of complications. These findings should therefore be interpreted as exploratory (**Figure 8**).

## 5 Discussion

In this prospective randomised trial, preoperative three-dimensional reconstruction improved several key intraoperative outcomes in uniportal VATS segmentectomy. Compared with conventional two-dimensional CT planning, 3D reconstruction shortened key-phase operative time, reduced intraoperative unexpected events, increased the rate of adequate pathological margin attainment, and improved surgeon-reported operative experience, without significantly affecting total operative time, postoperative recovery, or 30-day complications.

With respect to the time-related endpoints, key-phase operative time was significantly shorter in the 3D reconstruction group, whereas total operative time was similar between the groups. This pattern is biologically and procedurally plausible because 3D reconstruction is most likely to facilitate the core anatomical steps of segmentectomy, including hilar dissection, identification and management of the target bronchovascular structures, and delineation of the intersegmental plane. By contrast, total operative time also encompasses workflow-dependent components, such as waiting for frozen-section results, lymph node management, haemostasis and irrigation, drain placement, and chest closure, which are less likely to be influenced by preoperative visualisation. A time-saving effect in the same direction was also observed in complex segmentectomies. However, because the interaction test for procedural complexity was not statistically significant and the effect in simple segmentectomies did not reach statistical significance, these subgroup findings should be interpreted cautiously. Previous studies have reported heterogeneous findings regarding the effect of 3D reconstruction on total operative time, possibly reflecting differences in case mix and the limited sensitivity of total operative time to variation in non-critical procedural steps (15, 16, 24). Accordingly, key-phase operative time was prespecified as the primary time-based endpoint, excluding post-resection processes such as waiting for frozen-section results, lymph node dissection, and chest closure. This endpoint more directly captured the operative steps most likely to be influenced by 3D reconstruction, thereby improving the specificity and interpretability of the observed effect. These findings are plausible because 3D reconstruction translates axial CT data into an operative map, potentially reducing uncertainty regarding segmental anatomy and lesion depth (18, 19, 25).

Surgeon experience may have modified the observed effect of 3D reconstruction. The greater reduction in key-phase operative time among junior surgeons may indicate that patient-specific visualisation is particularly helpful when surgeons have less accumulated experience in mentally reconstructing complex segmental anatomy from conventional CT images. More experienced surgeons may already possess well-developed spatial interpretation skills and established operative strategies, leaving less scope for additional benefit from 3D visualisation. The reduction in intraoperative unexpected events was one of the most clinically meaningful findings of this study. During segmentectomy, such events are often related to unrecognised anatomical variations, vascular injury, or imprecise lesion localisation, particularly in complex procedures and in the presence of incomplete fissures (3, 9, 26). The distribution of individual events in our trial suggests that 3D reconstruction may primarily reduce anatomy- and localisation-related intraoperative difficulties. Erroneous division of a vessel or bronchus occurred only in the 2D CT group, and unplanned additional resection was less frequent in the 3D reconstruction group. By providing a patient-specific representation of bronchovascular anatomy and lesion location, 3D reconstruction may help surgeons identify relevant branches, anatomical variations, and potential margin limitations before the critical phase of hilar dissection, thereby reducing unexpected deviations from the planned procedure (27). Nevertheless, the composite outcome should be interpreted cautiously because its individual components differ in both mechanism and clinical importance. Vascular injury represents a direct intraoperative safety event; erroneous division represents an anatomy-related technical error; and unplanned additional resection primarily reflects difficulties in lesion localisation or margin planning. The observed reduction in the composite outcome appeared to be driven mainly by the absence of erroneous division and the lower frequency of unplanned additional resection in the 3D reconstruction group. However, the trial was not powered to compare the individual components separately, and the small number of events precludes definitive component-specific conclusions.

The improvement in adequate pathological margin attainment warrants particular emphasis. In segmentectomy, oncological adequacy depends not only on complete resection of the target segment but also on maintaining a safe distance between the tumour and the resection plane (28–30). Preoperative 3D planning may improve margin assessment by clarifying the spatial relationships among the tumour, the segmental hilum, and the intersegmental veins, which often serve as anatomical landmarks for the planned transection plane (10, 15, 31, 32). Our subgroup analyses further suggested that this margin-related benefit may be more pronounced among junior surgeons and in complex segmentectomies. Because this trial was designed to evaluate perioperative outcomes and the follow-up duration was limited, the higher rate of adequate pathological margin attainment should be interpreted as a finding related to surgical quality rather than evidence of improved long-term oncological control. The present data do not permit conclusions regarding recurrence, disease-free survival, or overall survival. In the short term, 3D reconstruction may also reduce the need for remedial measures prompted by margin concerns, such as additional resection triggered by frozen-section findings or subsequent intervention for a close final margin (9). This margin-related finding is clinically relevant because insufficient parenchymal margins and spread through air spaces have been associated with an increased risk of recurrence after limited resection (33–35).

No significant differences were observed in blood loss, chest tube duration, length of stay, or postoperative complications. These outcomes are likely to be influenced by multiple factors beyond preoperative imaging, including perioperative care pathways, postoperative pain control, air-leak management, and institutional discharge practices. In a minimally invasive setting such as uniportal VATS, the incremental effect of improved preoperative visualisation on short-term recovery may therefore be modest (24, 36).

In addition to objective clinical outcomes, surgeon-reported experience was evaluated. Surgeons in the 3D reconstruction group reported greater preoperative anatomical clarity, higher intraoperative confidence, and lower perceived stress. Collectively, these findings suggest that 3D reconstruction may reduce cognitive uncertainty and perceived stress during critical operative steps. Subgroup analyses further showed a greater reduction in key-phase operative time among junior surgeons, suggesting that 3D visualisation-guided planning may partly compensate for limited operative experience and potentially shorten the learning curve (37, 38). Surgeon-reported experience also favoured 3D reconstruction, consistent with literature highlighting the technical constraints of uniportal segmentectomy and the value of detailed planning, particularly for complex procedures (39).

This study has several limitations. First, its single-centre design may limit generalisability. Second, randomisation was not stratified by operating surgeon, and the statistical analyses were not adjusted for surgeon seniority. Residual confounding related to surgeon experience may therefore have influenced the estimated treatment effect. Third, although key-phase operative time was the prespecified primary endpoint, the sample size was estimated using total operative time because pilot measurements of the primary endpoint had not yet been standardised. This may have reduced the precision of the effect estimate. Fourth, follow-up was too short to assess long-term oncological outcomes. Finally, the surgeon-reported questionnaire was developed for this trial, lacked external validation, and was incomplete for some randomised procedures. Response bias and selection-related missingness therefore cannot be excluded, and these findings should be interpreted as exploratory. Longer follow-up is ongoing to assess the long-term oncological significance of margin attainment.

## 6 Conclusion

In conclusion, preoperative 3D reconstruction improved the efficiency and precision of uniportal VATS segmentectomy for early-stage NSCLC, with a greater apparent benefit among junior surgeons. Although the effect appeared larger in complex segmentectomies, the interaction was not statistically significant and should be interpreted as exploratory.

## Data Availability

The raw data supporting the conclusions of this article will be made available by the authors, without undue reservation.
