# Supplementary material for: Impact of patient-specific three-dimensional reconstruction on surgical planning and perioperative outcomes in early-stage lung cancer: a randomised clinical trial
Source: Front Oncol. 2026 Aug 21;16:1887152. doi: 10.3389/fonc.2026.1887152 (PMC13541431; doi:10.3389/fonc.2026.1887152)
Supplement: Supplementary file 1 [file Table1.docx]

Supplementary Material

Explanatory Notes on the Definitions of Selected Variables

1. Pleural adhesions were recorded by the attending surgeon after establishment of the thoracoscopic view and before hilar dissection. Adhesions were defined as fibrous connections between the parietal and visceral pleura that required adhesiolysis to achieve adequate surgical exposure (“present”); cases not requiring adhesiolysis were classified as “absent.”
2. Interlobar fissure development was assessed intraoperatively according to the Craig–Walker grading system and recorded by the attending surgeon before fissure handling; Grades 1–2 were categorized as “well developed,” and Grades 3–4 as “poorly developed.”
3. Surgeon seniority was prespecified according to both the professional rank of the primary operating surgeon and prior independent experience in anatomical segmentectomy. Surgeons with fewer than 20 previously completed anatomical segmentectomies were classified as junior, whereas those with 20 or more were classified as senior; professional rank was retained as an auxiliary descriptive variable. The 20-case threshold was selected on the basis of prior reports on the learning curve for thoracoscopic segmentectomy and our institutional training experience.
4. Segmentectomy complexity was categorized according to the number of intersegmental planes involved. Procedures involving one intersegmental plane were classified as simple, whereas those involving two or more planes were classified as complex. In practical terms, S6 is commonly considered a simple segmentectomy, whereas S9 and S10 are typically considered complex segmentectomies.
5. A total of 102 questionnaires were returned, representing 70.9% of the randomized cohort; 54 of 72 operations in the 2D group and 48 of 72 operations in the 3D group had completed questionnaires.The surgeon questionnaire was a study-specific instrument administered to the attending surgeon within 2 hours after the procedure and without external assistance. It was designed to capture three conceptually distinct domains: (1) clarity of preoperative anatomic understanding and planning, (2) confidence during key intraoperative steps, and (3) perceived intraoperative stress/workload. Items 1-3 and 4, 5, 6, 8, and 10 used 5-point ratings, with higher scores indicating greater clarity or confidence. Stress/workload items included both visual analogue scale (VAS) ratings from 0 to 10 and 5-point ratings, with higher scores indicating greater perceived stress or workload. Domain scores were calculated as the mean of the constituent items. The instrument had not been externally validated before this study; psychometric testing was therefore performed within the present cohort.

Exploratory factor analysis supported a 3-factor structure (KMO = 0.860; Bartlett’s test: χ² = 813.282, df = 105, P < 0.001), with a cumulative variance explained of 66.548%. The 3 domains were defined as preoperative anatomic understanding and planning clarity (items 1–3), confidence during key operative steps (items 4–8), and perceived intraoperative stress and workload (items 7–13). All 3 domains demonstrated good internal consistency, with Cronbach’s α coefficients of 0.801, 0.798, and 0.901, respectively. Because the questionnaire was developed specifically for this study, these psychometric findings should be interpreted as evidence of internal psychometric performance rather than external validation.

**Surgeon Subjective Experience Questionnaire**

English Translation

**Instructions:** This questionnaire is intended to investigate surgeons' subjective experience. It should be completed within 2 hours after the end of each operation by interviewing the primary surgeon, with responses recorded by the investigator. The questionnaire consists of three parts. Please rate each item below according to your actual experience during the operation.

Date Completed: _____ / _____ / _____

Surgical Group:□ 3D Reconstruction Group; □ Conventional Chest CT Group Seniority:

□ Junior Surgeon	□ Senior Surgeon

**Dimension 1. Clarity of Preoperative Anatomic Understanding and Planning**

1. During the preoperative assessment, how clearly did you understand the patient's pulmonary anatomy and tumor location?

□ 1 Completely unclear

□ 2 Slightly unclear

□ 3 Neutral

□ 4 Clear

□ 5 Very clear

2. How would you rate the difficulty of preoperative planning?

□ 1 Very difficult

□ 2 Somewhat difficult

□ 3 Moderate

□ 4 Easy

□ 5 Very easy

3. Do you think the preoperative imaging examinations (e.g., CT, 3D reconstructed CT) were sufficiently clear to help you formulate the surgical plan?

□ 1 Completely unclear

□ 2 Slightly unclear

□ 3 Neutral

□ 4 Clear

□ 5 Very clear

**Dimension 2. Confidence During Key Intraoperative Steps**

4. How confident were you during this step (identifying the artery)?

□ 1 Very unconfident; frequent hesitation

□ 2 Less confident; repeated confirmation needed

□ 3 Neutral; managed routinely

□ 4 Fairly confident; occasional confirmation

□ 5 Very confident; no additional confirmation needed

5. How confident were you during this step (identifying the bronchus)?

□ 1 Very unconfident; frequent hesitation

□ 2 Less confident; repeated confirmation needed

□ 3 Neutral; managed routinely

□ 4 Fairly confident; occasional confirmation

□ 5 Very confident; no additional confirmation needed

6. How confident were you during this step (identifying the vein)?

□ 1 Very unconfident; frequent hesitation

□ 2 Less confident; repeated confirmation needed

□ 3 Neutral; managed routinely

□ 4 Fairly confident; occasional confirmation

□ 5 Very confident; no additional confirmation needed

7. How confident were you during this step (resection-margin localization)?

□ 1 Very unconfident; frequent hesitation

□ 2 Less confident; repeated confirmation needed

□ 3 Neutral; managed routinely

□ 4 Fairly confident; occasional confirmation

□ 5 Very confident; no additional confirmation needed

8. How confident were you during this step (identifying the intersegmental plane)?

□ 1 Very unconfident; frequent hesitation

□ 2 Less confident; repeated confirmation needed

□ 3 Neutral; managed routinely

□ 4 Fairly confident; occasional confirmation

□ 5 Very confident; no additional confirmation needed

**Dimension 3. Intraoperative Stress and Workload**

9. Please indicate the level of stress you experienced at this stage (segmental hilar dissection) (0 = no stress, 10 = extreme stress).

□ 1 point

□ 2 point

□ 3 point

□ 4 point

□ 5 point

□ 6 point

□ 7 point

□ 8 point

□ 9 point

□ 10 point

10. Please indicate the level of stress you experienced at this stage (resection-margin localization) (0 = no stress, 10 = extreme stress).

□ 1 point

□ 2 point

□ 3 point

□ 4 point

□ 5 point

□ 6 point

□ 7 point

□ 8 point

□ 9 point

□ 10 point

11. Please indicate the level of stress you experienced at this stage (identifying the intersegmental plane) (0 = no stress, 10 = extreme stress).

□ 1 point

□ 2 point

□ 3 point

□ 4 point

□ 5 point

□ 6 point

□ 7 point

□ 8 point

□ 9 point

□ 10 point

12. Mental demand: To what extent did you need to repeatedly think through the anatomic structures during the operation (1 = very low, 5 = very high)?

□ 1 point

□ 2 point

□ 3 point

□ 4 point

□ 5 point

13. Compared with your previous segmentectomy procedures, how would you rate the overall stress level of this operation?(1 = markedly lower; 10 = markedly higher)

□ 1 point

□ 2 point

□ 3 point

□ 4 point

□ 5 point

□ 6 point

□ 7 point

□ 8 point

□ 9 point

□ 10 point
